# Method comparison and overview of refractive measurements in children: implications for myopia management

**DOI:** 10.1136/bmjophth-2023-001322

**Published:** 2024-03-01

**Authors:** Jonas Müller, Xiaoqin Chen, Arne Ohlendorf, Lihua Li, Siegfried Wahl

**Affiliations:** 1 Institute for Ophthalmic Research, Eberhard Karls University Tuebingen, Tuebingen, Germany; 2 Tianjin Eye Institute, Tianjin Eye Hospital, Tianjin, China; 3 Carl Zeiss Vision International GmbH, Aalen, Germany

**Keywords:** Optics and Refraction, Child health (paediatrics), Drugs, Vision

## Abstract

**Objective:**

This study investigated the agreement between objective wavefront-based refraction and subjective refraction in myopic children. It also assessed the impact of cyclopentolate and refraction levels on the agreement.

**Methods:**

A total of 84 eyes of myopic children aged 6–13 years were included in the analysis. Non-cycloplegic and cycloplegic objective wavefront-based refraction were determined and cycloplegic subjective refraction was performed for each participant. The data were converted into spherical equivalent, J_0_ and J_45_, and Bland-Altman plots were used to analyse the agreement between methods.

**Results:**

Linear functions were used to determine the dependency between the central myopic refractive error and the difference between the method of refraction (=bias). The influence of central myopia was not clinically relevant when analysing the agreement between wavefront results with and without cyclopentolate (comparison 1). The bias for wavefront-based minus subjective spherical equivalent refraction (comparison 2) was ≤−0.50 D (95% limits of agreement −0.010 D to −1.00 D) for myopia of −4.55 D and higher when cycloplegia was used (p<0.05). When no cyclopentolate was used for the wavefront-based refraction (comparison 3), the bias of −0.50 D (95% limits of agreement −0.020 D to −0.97 D) was already reached at a myopic error of −2.97 D. Both astigmatic components showed no clinically relevant bias.

**Conclusion:**

The spherical equivalent, measured without cycloplegic agents, led to more myopic measurements when wavefront-based refraction was used. The observed bias increased with the amount of myopic refractive error for comparisons 2 and 3, which needs to be considered when interpreting wavefront-refraction data.

**Trial registration number:**

NCT05288335.

**WHAT IS ALREADY KNOWN ON THIS TOPIC**

- While the effect of overcorrecting myopic children without cycloplegic agents and variations in bias among different refraction methods are well known, there is a lag of knowledge about the agreement between wavefront-based refraction and subjective refraction in myopic children as well as the influence from cycloplegic agents and the level of myopia on it.

**WHAT THIS STUDY ADDS**

- Bland-Altman analysis in combination with linear functions shows that there is a good agreement between wavefront-based and subjective refraction for low myopia, with an increasing bias towards higher myopia.

**HOW THIS STUDY MIGHT AFFECT RESEARCH, PRACTICE OR POLICY**

- Understanding the level of myopia, to which good agreement between wavefront-based refraction and the clinical standard of subjective refraction can be assumed, is particularly important for epidemiological studies and practical myopia care.

## Introduction

Myopia has become a major health issue,^1^ especially among children and adolescents in Asian countries^2 3^ over the last decades. Studies have revealed an average prevalence of 60% of myopia in western China, among children and adolescents between 6 and 21 years.^4^ Additionally, home confinement as a result of the COVID-19 pandemic accelerated the reported prevalence and progression of this refractive error.^5^ A comparison of prevalence before and after the pandemic indicates an increase in prevalence from 48.2% to 60.0% among the 7–18 years across the country.^5^ It is well known that the younger the affected children are at the onset of myopia, the higher levels of myopia are expected at older ages^6^ and this can have adverse effects on ocular health. The increased growth of myopic eyes in case of axial myopia not only increases the risk of developing several pathologies^7^ but also poses a significant threat to vision as these diseases can lead to blindness.^8^

To address the issues myopia can have on individual ocular health and economic burdens,^9 10^ various myopia management solutions have been developed and clinically investigated and are now available.^11^ Primary outcome measures in clinical research and real-world evidence of myopia management solutions are the progression of axial length and the refractive error of the eye.^12 13^ The International Myopia Institute has established recommendations^12–16^ for screening, diagnosis and management of myopia in children and adolescents. In case of refractive errors, it is recommended to use an open-field autorefractor under cycloplegic conditions, especially for clinical studies.^13^ While such recommendations are appreciated to allow a standardised way on how such clinical data is obtained, real-world myopia management often does not include the use of pharmaceutical agents such as atropine/tropicamide to relax accommodation of the crystalline lens and additionally, a variety of refraction methods (objective/subjective) are used.^17^ The literature indicates that different results can be expected between objective measurement methods and subjective refraction, especially when different devices for the measurement of the refractive error are used.^18–23^ To conclude, there is a gap between the best clinical practice as described in standards and how ophthalmologists and optometrists work in their daily practice when conducting myopia management. In order to be able to interpret refractive error data in the myopia management practice that are either obtained with or without the use of cycloplegic agents, it is crucial to understand the limits of subjective and objective refractive error measurements when obtained under different circumstances. The aim of the current study was to deepen the understanding of the agreement of different methods of assessing refractive error that are also mixed in the daily practice. Agreement of spherocylindrical refractive errors in a cohort of myopic children will be assessed using cycloplegic and non-cycloplegic wavefront-assisted objective refraction as well as cycloplegic subjective refraction.

## Methods

### Inclusion and exclusion criteria

The inclusion criteria were defined as the cycloplegic objective spherical refractive error between −0.75 D and −5.00 D and for a cylinder of ≤1.50 D (rounded to 0.25 D). In addition, anisometropia was not allowed to exceed 1.50 D and best-corrected visual acuity ≤0.0 logMAR was required. Primary exclusion criteria included ocular trauma or surgery, ocular pathologies and systemic diseases affecting the immune system. Also excluded were children with elevated intraocular pressure (>21 mm Hg or a difference of both eyes of ≥5 mm Hg) and subjects undergoing myopia control management methods.

### Procedures

The protocol contained medical history taking, intraocular pressure measurement, slit lamp and fundus examination, and unilateral cover test. Additionally, objective wavefront-based and subjective refraction were performed in both eyes and visual acuity was checked with a decimal visual acuity chart. Cycloplegia was induced by three drops of 1% cyclopentolate, 5 min apart and measurements were started at the earliest 30 min after application of the last drop. For verification that cycloplegia was achieved, it was ensured that no pupillary reflex was detectable anymore. In contrast, the International Myopia Institute recommends as a frequently used procedure to apply either two drops of 1% tropicamide separated by 5 min and to start 30 min postapplication.^13 24^

An aberrometry-based device was used to measure objective refraction before and after the application of cyclopentolate (ZEISS I.PROFILER PLUS; CARL ZEISS VISION, Germany). Subjective refraction was measured only under cycloplegic conditions using a phoropter (VT-10; Topcon, Japan) and a visual acuity chart. The endpoint of the subjective measurement of refractive errors was defined as the maximum plus lens that achieved a visual acuity of ≤0.0 logMar.

### Refractive error comparisons

To compare refractive error measurements, analysis is focused on methods of refractive error measurement where only one variable is changed at the time (comparisons #1 and #2). In order to reflect the already described potential clinical scenario, two variables in comparison #3 were changed.

In the present study, a total of three comparisons have been conducted:

1. Non-cycloplegic objective and cycloplegic objective refraction.
2. Cycloplegic objective and cycloplegic subjective refraction.
3. Non-cycloplegic objective and cycloplegic subjective refraction.

### Statistical analysis

To prepare for the analysis, the refraction results were converted into power vectors (SE=spherical equivalent, J_0_=orthogonal cylinder component, J_45_=oblique cylinder component) using the equations previously described in the literature.^25^ The distribution of data was analysed using Kolmogorov-Smirnov test and Q-Q plots to evaluate if data have been normally distributed. The agreement between different refractive error measurements (#1 non-cycloplegic objective and cycloplegic objective refraction; #2 cycloplegic objective and cycloplegic subjective refraction and #3 non-cycloplegic objective and cycloplegic subjective refraction) has been analysed by using Bland-Altman analysis to further visualise and evaluate differences between the methods (=bias).^26 27^ To account for the correlation between both eyes, only the right eye was included in all analyses and the statistics were conducted with R V.4.2.2.

## Results

### Sample characteristics

Eighty-four myopic children (median objective cycloplegic spherical equivalent of the right eye: −2.54 D IQR −1.79 D to −3.61 D) were included in the study. The study group consisted of 52 boys and 32 girls aged between 6 and 13 years (mean age: 10.13±1.59 years) at the beginning of the study.

### Bland-Altmann analysis

**Figure 1:**
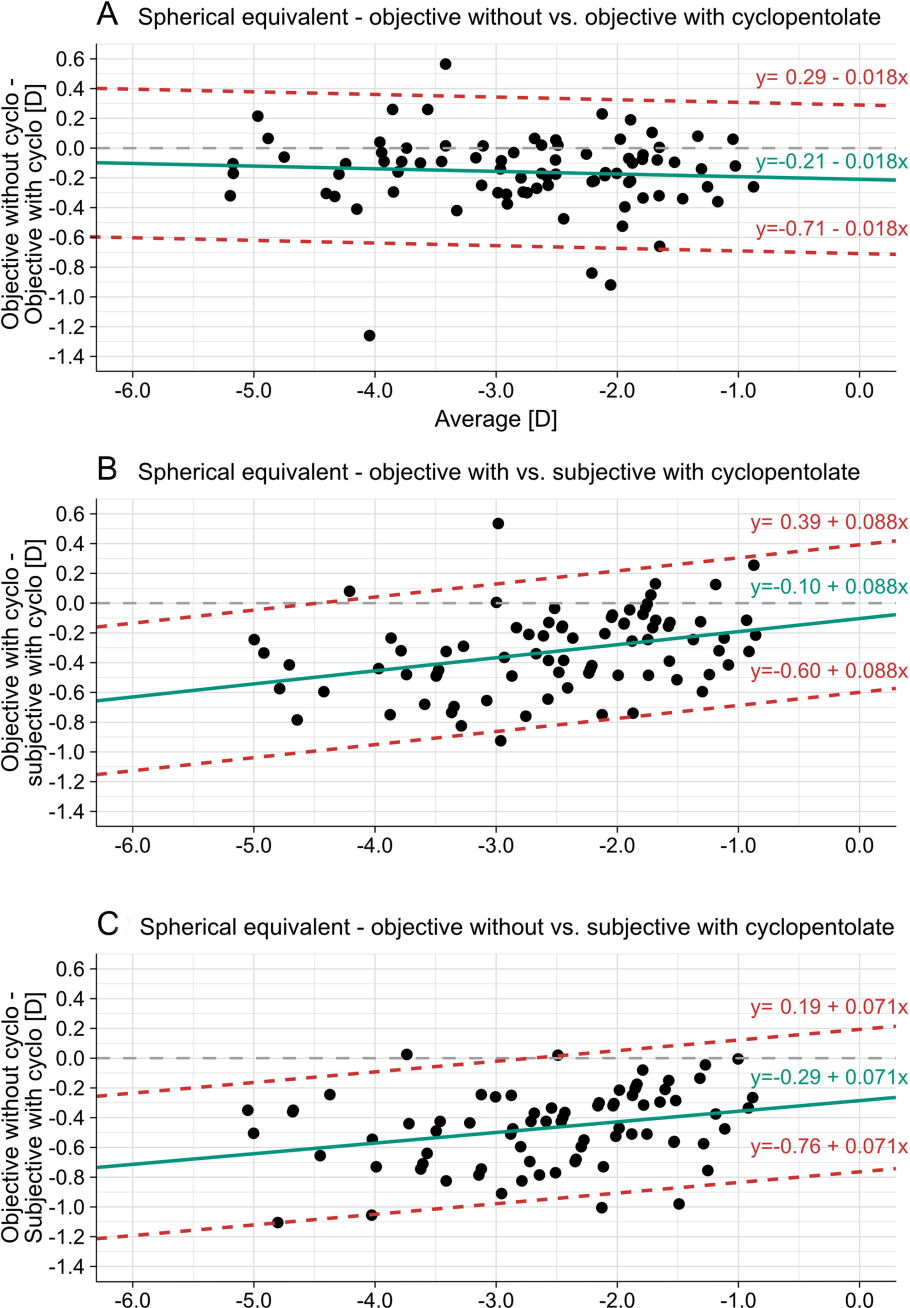
Bland-Altman plots for spherical equivalent: red lines display LOAs, green lines depict bias and grey dashed lines represent points of no differences between both methods. LOAs, Limit of agreements.

‘Traditional’ Bland-Altmann analysis assumes a normal distribution of data, which was not given in the present data set. Consequently, the modified approach proposed by Bland and Altman^27^ was employed and linear regression (y= β_0_+β_1_x_1_) was used for determining the bias between the different comparisons of measurements of refractive errors and can be found in table 1 and in figure 1.

**Table 1:** Overview for agreement between the different measurement conditions

	Non-cycloplegic objective and cycloplegic objective refraction (comparison 1)	Cycloplegic objective and cycloplegic subjective refraction (comparison 2)	Non-cycloplegic objective and cycloplegic subjective refraction (comparison 3)
**SE**	Bias: y=−0.21–0.018 x (LOA: y=0.29–0.018 x to y=−0.71–0.018 x) P(β_1_)=0.49	Bias: y=−0.10+0.088 x (LOA: y=0.39+0.088 x to y=−0.60+0.088 x) P(β_1_)=0.05	Bias: y=−0.29+0.071 x (LOA: y=0.19+0.071 x to y=−0.76+0.071 x) P(β_1_)<0.05
	MD −0.16 (95% CI 0.17 to −0.49)	MD −0.33 (95% CI −0.0057 to −0.65)	MD −0.49 (95% CI −0.17 to −0.81)
**J_0_**	Bias: y=0.0049–0.086 x (LOA: y=0.22–0.086 x to y=−0.21–0.086 x) P(β_1_)=0.12	Bias: y=−0.041+0.37 x (LOA: y=0.20+0.37 x to y=−0.28+0.37 x) P(β_1_)<0.05	Bias: y=−0.047+0.31 x (LOA: y=0.19+0.31 x to y=−0.29+0.31 x) P(β_1_)<0.05
	MD 0.020 (95% CI 0.045 to −0.085)	MD 0.017 (95 % CI 0.085 to −0.051)	MD −0.0031 (95 % CI 0.061 to −0.067)
**J_45_**	Bias: y=−0.0045+0.034 x (LOA: y=0.17+0.034 x to y=−0.18+0.034 x) P(β_1_)=0.64	Bias: y=0.020+0.31 x (LOA: y=0.18+0.31 x to y=−0.14+0.31 x) P(β_1_)<0.05	Bias: y=0.027+0.35 x (LOA: y=0.20+0.35 x to y=−0.15+0.35 x) P(β_1_)<0.05
	MD −0.0063 (95% CI 0.036 to −0.049)	MD 0.0088 (95% CI 0.052 to −0.034)	MD 0.0025 (95 % CI 0.046 to −0.041)

LOA, limit of agreement; MD, mean difference.

Analysing the agreement of different methods during assessment of refractive errors objectively/subjectively as well as with or without the use of cycloplegic agents has revealed the following: for the comparison 1 (non-cycloplegic vs cycloplegic objective refraction) has a small, clinical not significant mean difference and available data did not revealed an clinical relevant influence on the amount of the central myopic refractive error for the spherical equivalent refractive error. In case the method of refraction is changed, but pharmaceutical state of the eye stays similar (comparison 2, cycloplegic objective and cycloplegic subjective refraction) leads to a higher mean difference (meaning that the cycloplegic subjective measurement of the spherical equivalent is more positive compared with the situation when refractive errors are measured objectively). Additionally and different to comparison 1, the amount of central myopic spherical equivalent refractive error showed a significant influence of the central error, meaning that agreement is reduced with increasing levels of myopia. The very same results have been registered for comparison #3, when non-cycloplegic objective measurement and cycloplegic subjective refraction are compared, also the mean difference revealed clinically relevant differences for the spherical equivalent refractive error.

For the two astigmatic components, the results are similar for comparison 1. The influence of the refractive error on the bias is not significant and the mean difference shows a negative bias and thus a slight myopic overcorrection without cycloplegia.

Comparisons 2 and 3 indicate a similar regression with a statistically significant influence of the height of the refraction. However, it only becomes clinically relevant with higher cylinders (eg, J_0_>0.4). This is also reflected in the mean differences for both components and comparisons, which are not clinically relevant.

## Discussion

### Agreement for spherical equivalent: non-cycloplegic objective refraction versus cycloplegic objective refraction

**Figure 2:**
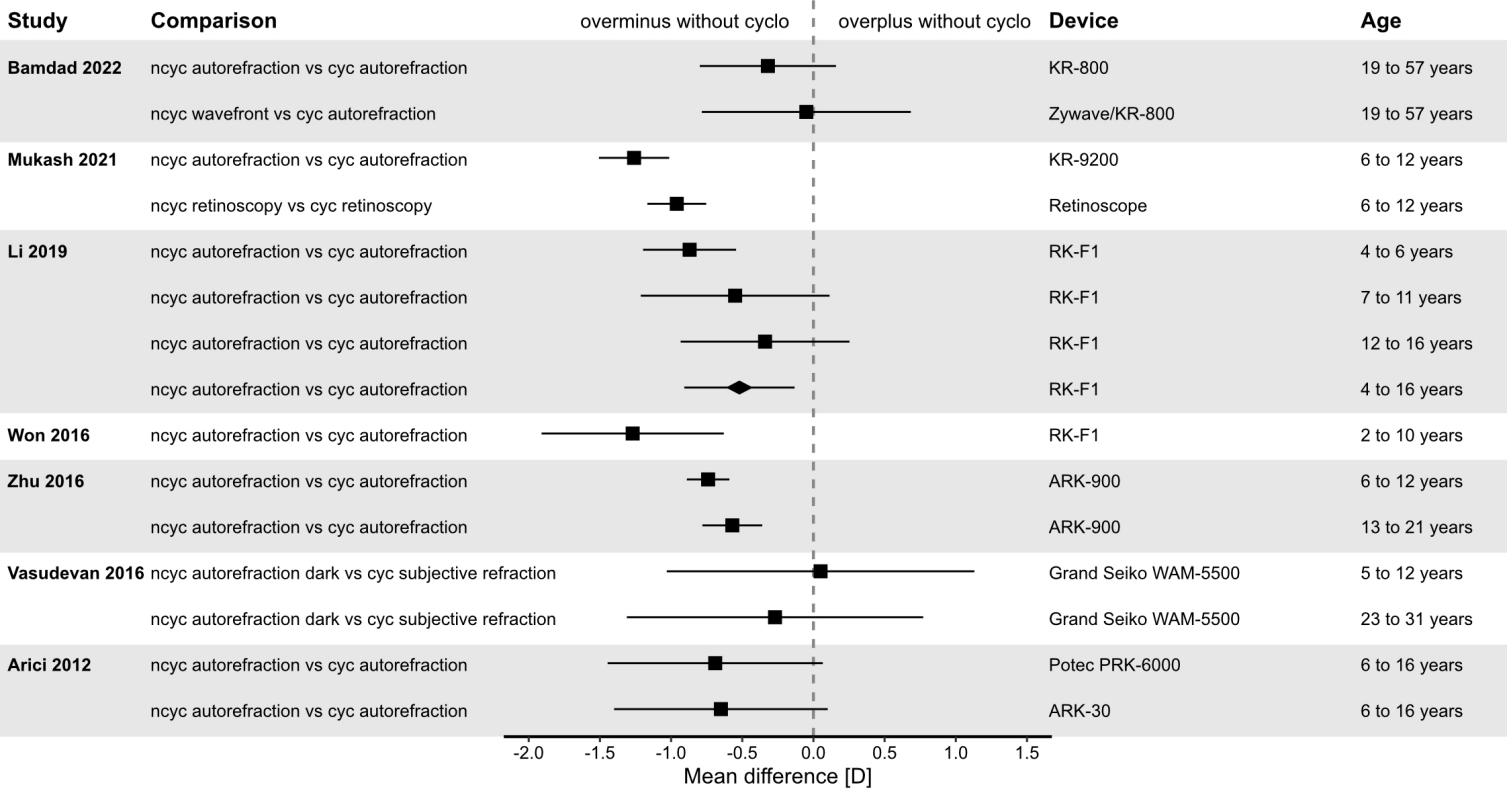
Forest plot with bias and limits of agreement separated by age and measurement method. cyc, cycloplegic; ncyc, non-cycloplegic.

**Figure 3:**
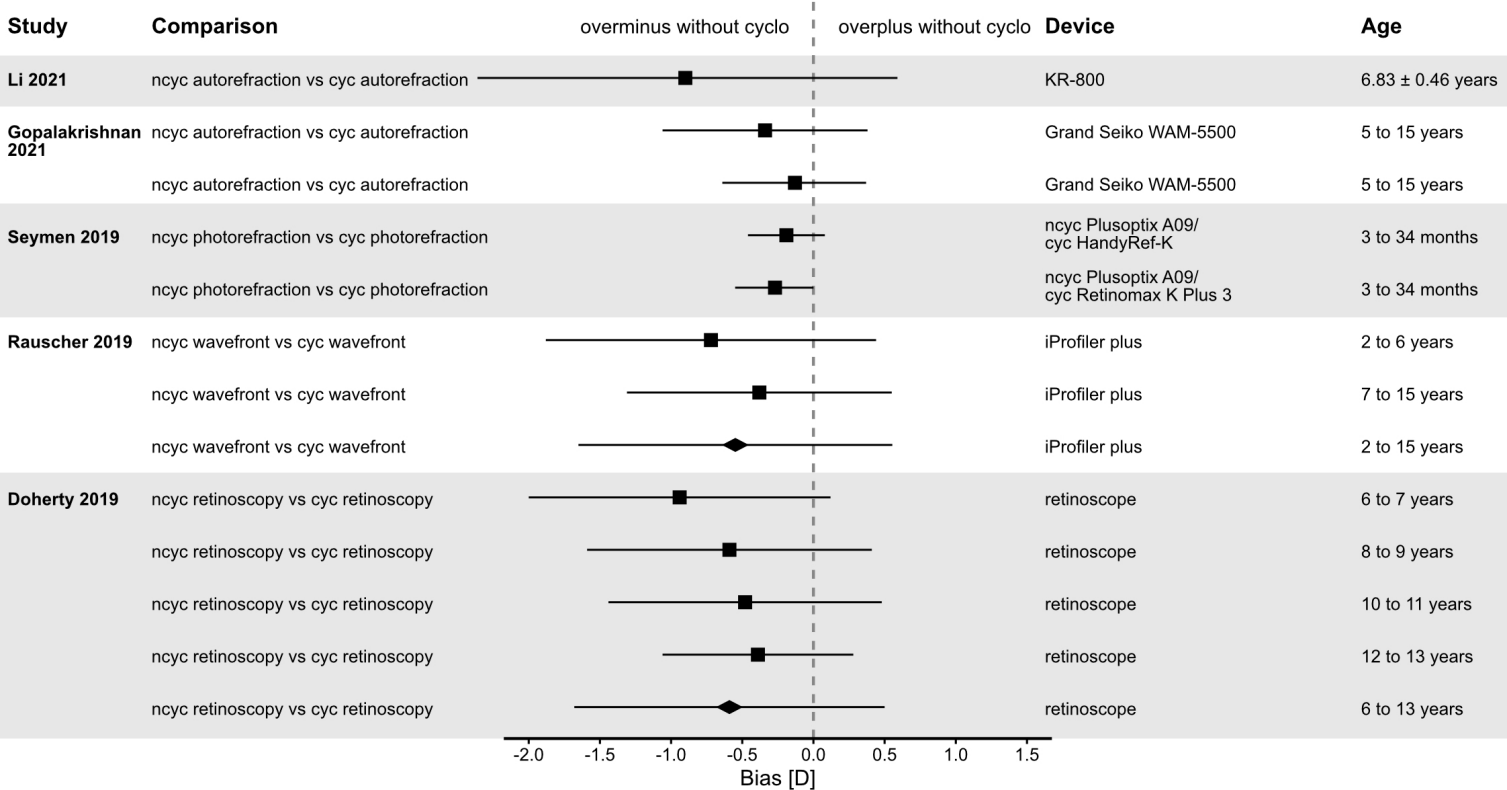
Forest plot with mean difference and 95% CIs separated by age and measurement method. cyc, cycloplegic; ncyc, non-cycloplegic.

To put the results of the presented research in a ‘historical’ context, forest plots have been created to give an overview of previous literature looking into comparable agreement comparisons (see figures 2 and 3). The results of the current study indicate that for the spherical equivalent, there is a risk of myopic overcorrection without the use of cyclopentolate. This is well known for a variety of measurement techniques that do not require the direct cooperation of the subject, such as autorefraction,^18 19 28–34^ retinoscopy^28 35^ and photorefraction.^36 37^ A similar trend can be observed for wavefront-assisted objective refraction. The literature has documented undercorrection in hyperopic children without the use of medication to block accommodation.^38 39^ In case of myopic children, overcorrection with minus was demonstrated in a small sample (n=10), confirming our study for a larger population.^40^

In summary, the results for comparison #1 are consistent with the literature and the effect of cycloplegic agents on wavefront-assisted refraction is comparable to other measurement methods.

### Agreement for spherical equivalent: cycloplegic objective refraction versus cycloplegic and non-cycloplegic subjective refraction

In case the agreement between cycloplegic or non-cycloplegic wavefront-based refraction and cycloplegic subjective refraction (comparisons #2 and #3) is analysed, analysis shows a dependency of the bias on the central (myopic) refractive error, a result that has not been significant when analysing the intra-agreement (#comparison #1).

As can be noted from previous research, the spread between objective and subjective assessment has been reported to be quite high.^22 23^ Additionally, aberrometry-based autorefractors seem to have a better accuracy^21^ and a similar agreement to traditional autorefractors.^18^ In contrast, Bennett *et al* demonstrate a slightly better agreement between autorefractors and subjective refraction than wavefront-based systems and subjective refraction. However, this difference does not reach clinical relevance either in the mean bias or in the LOAs.^20^

Contrary to our finding with cyclopentolate (comparison #2), Bamdad *et al* reported a non-statistically significant difference in an adult population between subjective refraction without cycloplegia and wavefront-based refraction without cycloplegia. However, by consulting their agreement analysis, it is clear that their reported differences (SE-LOAs: +0.73 D to −0.81D) can be clinically significant.^18^

In case of comparison #3, it is still questionable if the observed lower agreement especially for high myopia is caused by (1) the used method (aberrometry versus subjectiv refraction), (2) the use of a cycloplegic agent or (3) the combination of both. To understand this more precisely, the results of comparison #2 must be taken into account, as this comparison revealed not only a significant difference between both tested methods but also a dependency of the results on the amount of the central refractive error. In turn, parts of the observed difference in comparison #3 might be caused by the fact that aberrometry-based autorefraction was used, rather than through the use of a cycloplegic agent. However, as the use of such agents reduces the deviation of the data (smaller offset, see comparison #2). In contrast, the dependence on the amount of central refractive error remains unaffected by cycloplegia (slope in comparisons #2 and #3 similar) and is most likely caused by the use of the different methods, while the influence of the cycloplegic agent might be rather small. In summary, it can be stated that part of the offset is probably due to the parameter cycloplegia (non-cycloplegic vs cycloplegic) and the slop is due to the different measurement methods (aberrometry versus subjective refraction).

For the observed dependency of the results on the central refractive error, the literature does not show a clear picture. Cooper *et al* compared a traditional autorefractor and an aberrometer with subjective refraction. Figure 4 of Cooper *et al* reveals an increase in bias with increasing myopia. However, this only affects the wavefront refraction and not the classical autorefraction and is, therefore, consistent with our findings and assumptions.^21^ Contrary to this, Bamdad *et al* and Bennett *et al* could not find such a relationship.^18 20^ Nevertheless, in contrast to our study, both studies were conducted on adults. Bennette *et al* did not use the spherical equivalent but a vector describing all three refraction components to calculate the bias. In addition, they perform a logarithmic transformation of the results to generate a normal distribution.^20^ This results in the slope of a linear regression being influenced by this transformation and a comparison is no longer possible.^41^

The only study to date (besides a poster presentation with low sample size^40^) that was also performed with the ZEISS I.PROFILER PLUS found a comparable bias of −0.55 D in the spherical equivalent with limits of agreement ranging from 0.55 to −1.65 D. These results correspond to all comparisons determined in the present study and is, therefore, also in agreement for a purely myopic population of children.^38^ For a more detailed comparison of the bias, an estimate can be made by applying the mean refraction of the study by Rauscher *et al* to our linear regression function (comparison #3). According to the predicted results, this leads to a bias of −0.19 D, which is within the LOAs of the previous study and in line with other LOAs in the literature for other measurement methods.^29 30 35 37 38^

### Agreement for astigmatic components

Consistent with the literature, this research has found that the astigmatic components are influenced to a lesser extent by cycloplegic agents.^31 34 38^ A relevant difference in both components only occurs when objective refraction is compared with subjective refraction and this is also consistent with the results already discussed for the spherical equivalent. As for the spherical equivalent, this deviation is mainly the result of the consideration of the refraction level and must be taken into account accordingly.

### Limitations

The findings of this study are subject to at least three main limitations. First, the comparison between objective and subjective refraction may be influenced by the different cooperation needed from the participants depending on the measurement methods and the different cooperation levels of the children during the subjective refraction. The subjective refraction is also limited by the step size of 0.25 D, which may affect the bias results since the objective refraction uses a step size of 0.01 D. Second, it remains unclear whether the relationship between the bias and the amount of myopia applies to higher degrees of myopia beyond the range present in the sample population. Third, using a phoropter to examine children can be challenging as decentration can occur. Although a phoropter has been used for cycloplegic subjective refraction^42^ in a comparable age range, methods were taken to keep the influence low. Therefore, to avoid this influence, special attention was paid to a correct centration during the entire measurement and if necessary, the centration was improved.

## Conclusions

The aim of the current study was to assess the agreement between cycloplegic and non-cycloplegic wavefront-assisted objective refraction as well as cycloplegic subjective refraction, in order to assist the interpretation of refractive error measurements, especially in myopia management. The intra-agreement between the objective comparisons was observed to be high. The results indicate that the amount of myopia is crucial for the bias between wavefront-assisted refraction and subjective refraction, and therefore, needs to be taken into account. This effect is independent of the cycloplegic agent and consequently the cause is likely to be the different measurement technique.

By applying the obtained functions, it can be determined at which point a predefined bias is reached and the methods of predefined parameters can no longer be regarded as sufficiently in agreement. For a bias (objective cycloplegic vs subjective cycloplegic) of −0.50 D (LOA: −0.010 D to −1.00 D), this value is reached at a refraction of −4.55 D. If the variable cycloplegia is also changed, the −0.50 D (LOA: −0.020 D to −0.97 D) bias is already reached at −2.97 D.

These findings provide insights into the relationship between wavefront-based autorefraction and subjective refraction, considering the use of cycloplegia in myopic children. This enables data sets obtained using wavefront-based autorefractors to be interpreted and, for instance, complement epidemiological studies in the future. As a result, this can improve the exchange and communication between clinical research and practical myopia care. Therefore, this is relevant to both eye care practitioners and clinical scientists in the field of myopia.

## Data Availability

Data are available on reasonable request.
